# Implementation opportunities and challenges identified by key stakeholders in scaling up HIV Treatment as Prevention in British Columbia, Canada: a qualitative study

**DOI:** 10.1186/s43058-020-00044-2

**Published:** 2020-06-16

**Authors:** Koharu Loulou Chayama, Ryan McNeil, Jean Shoveller, Will Small, Rod Knight

**Affiliations:** 1 British Columbia Centre on Substance Use, 400-1045 Howe Street, Vancouver, BC V6Z 2A9 Canada; 2 Department of Experimental Medicine, Faculty of Medicine, University of British Columbia, 2775 Laurel Street, Vancouver, BC V5Z 1 M9 Canada; 3 General Internal Medicine, School of Medicine, Yale University, 333 Cedar Street, New Haven, CT 06520 USA; 4 Yale Program in Addiction Medicine, School of Medicine, Yale University, 333 Cedar Street, New Haven, CT 06520 USA; 5 Department of Community Health and Epidemiology, Faculty of Medicine, Dalhousie University, 1459 Oxford Street, Halifax, NS B3H 4R2 Canada; 6 Izaak Walton Killam Health Centre, 5850/5980 University Avenue, Halifax, NS B3K 6R8 Canada; 7 Faculty of Health Sciences, Simon Fraser University, Blusson Hall, Room 11300, 8888 University Drive, Burnaby, BC V5A 1S6 Canada; 8 Centre for Applied Research in Mental Health and Addiction, Faculty of Health Sciences, Simon Fraser University, 515 W. Hastings Street, Vancouver, BC V6B 5 K3 Canada; 9 Department of Medicine, Faculty of Medicine, University of British Columbia, 317-2194 Health Sciences Mall, Vancouver, BC V6T 1Z3 Canada

**Keywords:** HIV, Treatment as Prevention, Consolidated Framework for Implementation Research, Canada

## Abstract

**Background:**

The province of British Columbia (BC), Canada, was among the first jurisdictions to scale up HIV Treatment as Prevention (TasP) to the population level, including funding and policy commitments that enhanced HIV testing efforts (e.g., expansion of routine, opt-out testing), while also making antiretroviral therapy universally available to all people living with HIV. As such, BC represents a critical context within which to identify factors that influenced the scalability of TasP (e.g., acceptability, adoption, fidelity, equitable reach, sustainability), including key opportunities and challenges.

**Methods:**

We draw on in-depth, semi-structured interviews with 10 key stakeholders, comprised policymakers at the local and provincial levels and representatives from community-based organizations. Using the Consolidated Framework for Implementation Research (CFIR) to guide data collection, coding, and analysis, we identified key factors that influenced practice transformation and scale up.

**Results:**

Key factors that contributed to the successful scale up of TasP included: (i) opportunities that enhanced stakeholder buy-in based on features of the *intervention characteristics*, including with regard to assessments about the quality and strength of evidence supporting TasP; (ii) an *inner setting* implementation climate that was, in part, shaped by the large and highly symbolic government investments into TasP; (iii) features of the *outer setting* such as external policies (e.g., harm reduction) that cultivated opportunities to implement new “systems-level” approaches to HIV intervention; (iv) the personal attributes of some “middle-level” influencers, including a team that was comprised of some highly motivated and social justice-oriented individuals (e.g., folks who were deeply committed to serving marginalized populations); and (v) the capacity to develop various *implementation processes* that could maintain “nimble and evidence-informed” adaptations across a highly decentralized service delivery system, while also creating opportunities to adapt features of TasP programming based on “real time” program data.

**Conclusion:**

Constructs across all five domains of CFIR (intervention characteristics, outer setting, inner setting, characteristics of individuals, and process) were identified to influence the success of TasP in BC. Our findings provide important insights into how BC can successfully implement and scale up other systems-level interventions that have demonstrated efficacy, while also offering insights for other jurisdictions that are currently or planning to scale up TasP.

Contributions to the literature

- Implementation gaps exist in understanding the factors that influence the scale-up of Treatment as Prevention (TasP), a strategy increasingly adopted worldwide to reduce HIV transmission.
- Using the Consolidated Framework for Implementation Research, this study identified the key implementation factors that influenced the scale-up of TasP in British Columbia (BC), Canada—one of the first jurisdictions to scale up to the population level.
- Our findings provide critical insights for other jurisdictions that are currently scaling up TasP, as well as offer contextualized understandings about how BC can effectively go to scale with other promising population-level interventions.

## Introduction

During the past decade, the role of antiretroviral therapy (ART) in preventing HIV has had a critical impact in reducing the transmission of HIV and reshaping the broader HIV continuum of care [1]. Building on ecological data from observational research in the mid to late 2000s [2–4] in which the preventive benefit of ART was described, the landmark clinical trials HPTN-052 [5] and Partners of People on ART—A New Evaluation of the Risks (PARTNER) [6, 7] established during the early to late 2010s and strengthened recently with new data that ART confers a protective benefit against HIV transmission among both heterosexual and male homosexual serodiscordant couples. Clinical research, including the Strategic Timing of Antiretroviral Therapy (START) trial [8], has further established that the “early” initiation of ART (e.g., when a patient’s CD4+ T cell count is still high) also provides protective benefits at the individual level, including lower risks of developing AIDS and non-AIDS-related conditions (e.g., cardiovascular disease, non-AIDS-related cancers) [9].

Today, the universal testing and treatment strategies implemented to achieve viral suppression among people living with HIV (PLHIV) and to reduce risk of onward HIV transmission—referred to as “Treatment as Prevention” (TasP) and based on the premise and scientific consensus that undetectable viral loads are untransmittable or “undetectable equals untransmittable” (U=U)—is increasingly relied upon as a critical component of combination approaches to addressing the HIV epidemic [10, 11]. For example, in 2014, the UNAIDS adopted the “90-90-90” testing, treatment, and viral load suppression targets [12], an approach that emphasizes the need to meet the following targets by 2020: (i) 90% of all PLHIV will know their HIV status; (ii) 90% of all people diagnosed with HIV infection will receive ART that includes the use of antiretroviral drugs; and (iii) 90% of all people receiving ART will achieve viral suppression.

The province of British Columbia (BC), Canada, was among the first jurisdictions globally to begin implementing TasP and scale up the approach to the population level through its Seek and Treat for Optimal Prevention of HIV/AIDS (STOP HIV/AIDS) Project [13–15]. Since 2010, BC’s provincial funding and policy commitments have supported the TasP scale-up through the following: (i) enhanced efforts to test widely (e.g., via the implementation of voluntary routine testing guidelines across all primary and acute care settings, targeted/outreach testing campaigns); (ii) treat all clinically eligible PLHIV via the universal availability of ART for PLHIV and the “earlier” initiation of ART (i.e., as soon as possible following seroconversion and regardless of an individual’s CD4+ count); and (iii) strategies to ensure PLHIV achieve and maintain optimal ART adherence and viral suppression, including through intensive case management by the STOP Outreach Team—an interdisciplinary team of nurses, outreach workers, social workers, and administrative support workers that works with PLHIV to address barriers to engagement in HIV treatment and care, including those related to the social determinants of health (e.g., housing, food security) [15–17].

In 2010, STOP HIV/AIDS was initially implemented as a pilot in Vancouver and Prince George, two of the worst affected regions in the province, and supported by the BC government’s funding commitment of $48 million CAD over 4 years [18]. Following the success of the pilot, additional annual funding of $19.9 million CAD was invested by the BC government and STOP HIV/AIDS was expanded province-wide in 2013 [18]. Recent research examining the impact of TasP on the HIV epidemic in BC reported that HIV testing rates have more than doubled from 262 in 2009 to 611 in 2017 per 1,000,000 population [19]. Importantly, increases in the proportion of viral load suppression as a result of ART expansion and retention efforts have been achieved at both the individual and community levels, with a dramatic decrease from nearly 50% of participants with viral load ≥ 1000 copies/mL in 2006 to 10% in 2017 [19]. Since the province-wide expansion of TasP, the incidence of HIV has been declining across all regions of BC and among all exposure categories used in the 2017 HIV/AIDS Report by BC Centre for Disease Control, including among gay, bisexual, and other men who have sex with men, people who inject drugs, heterosexual contact, and blood, occupational, perinatal, and/or other exposures [20]. These reductions in HIV incidence likely reflect the success of TasP expansion, but it is important to point out that these reductions in HIV incidence likely also reflect other sustained prevention initiatives (e.g., syringe distribution programs, supervised injection sites) within some risk categories [20].

As various jurisdictions scale up TasP to address the HIV/AIDS epidemic and respond to the UNAIDS 90-90-90 targets, many of the key implementation factors that influence scale-up of TasP remain undocumented. As global progress towards achieving the 90-90-90 goals slows and remains uneven across regions, identifying the key factors that influence the scale-up of TasP may hold critical insights that can help jurisdictions that are currently implementing and scaling up TasP. Furthermore, identifying the implementation factors that have influenced scale-up within jurisdictions that have successfully scaled up TasP to the population level may also provide local insights into how new and emerging systems-level population health interventions with demonstrated efficacy (e.g., direct-acting antiviral treatments for hepatitis C virus, combination approaches to respond to North America’s opioid overdose crisis) can be effectively scaled up. As such, BC represents a critical implementation context within which to identify the factors that influenced the implementation and scalability of TasP, including the key opportunities and challenges [21].

### Objective

Using the Consolidated Framework for Implementation Research (CFIR), the current study was undertaken to identify key implementation opportunities and challenges that key stakeholders described as having influenced the implementation and scale-up of TasP in BC [22].

## Methods

### Conceptual framework

To identify a systematic and comprehensive understanding of the key implementation factors that influenced the implementation, adaptation, and scale-up of TasP, we draw on CFIR. The CFIR is well suited to guide an evaluation of the implementation of TasP as it provides a comprehensive framework to systematically identify the implementation factors that may emerge across contexts to influence a suite of implementation outcomes. Specifically, the CFIR features a “menu of constructs approach” [23], where the focus includes those factors relevant to the specific context and intervention of study. In the current study, we used CFIR in data analysis to identify the key implementation factors that influenced the implementation and scale-up of TasP. Specifically, we identify how the following intervention constructs featured within the experiences and perceptions of key stakeholders involved in various capacities (e.g., as implementation decision-makers) during and throughout scale-up:

1. Intervention characteristics, which include measurement of key features of TasP that influence implementation (e.g., stakeholders’ perceptions about the relative advantage of implementing TasP, complexity)
2. Inner setting, which includes contextual features internal to TasP that influence implementation (e.g., implementation climate, leadership engagement)
3. Outer setting, which includes contextual features external to TasP that influence implementation (e.g., external policies and incentives)
4. Characteristics of individuals, which focus on the individuals (internal and external stakeholders) involved in implementation and those who influence implementation (e.g., stakeholder knowledge, beliefs and assumptions about TasP)
5. Implementation process, which includes strategies or tactics that influence implementation (e.g., engaging appropriate individuals in the implementation and use of TasP, reflecting, and evaluating)

### Interviews

We draw on semi-structured, in-depth individual interviews with ten key stakeholders, including policymakers and representatives from community-based organizations. Specifically, participants were recruited through stratified purposeful sampling approach to select a sample that included a variety of viewpoints with specific attention to stratification across governmental institutions (e.g., at the local and provincial levels), non-governmental organizations, and different areas of expertise [24]. Among those invited to participate in the study, one declined participation due to time constraint and three could not be scheduled due to scheduling conflicts. The interviews took place in our private research offices, at the participants’ private office spaces, or over the phone. Each interview was recorded with a digital voice recorder and lasted an average of 1 h. Our study received ethics approval from Simon Fraser University’s Research Ethics Board (2014 s0555).

Before beginning the interview, participants provided a written or e-signature informed consent. Interviews were conducted by an experienced researcher (senior author RK). Recognizing that participants may be reluctant to share specific experiences and attitudes, RK provided assurances of privacy and confidentiality prior to the interviews. Interviews concentrated on the participants’ historical perceptions of and experiences with TasP, including those during the early implementation and scale-up phases. Our semi-structured interview guide included questions that asked participants to discuss their role with implementing and scaling up TasP, and we probed participants about the various challenges and facilitators that they experienced in those roles. For example, we asked participants about how the implementation of TasP was perceived to be influenced by a variety of factors, including their own roles within their respective organizations, recognizing that most participants’ roles changed as TasP was scaled up during the past decade. Finally, we asked a series of questions to elicit the implementation factors that participants felt may have influenced the success of TasP to scale up across BC, including with respect to key individual and/or institutional “influencers” (e.g., decision and policymakers, regulatory bodies) and structural-level facilitators or challenges (e.g., factors that influenced the scale-up of TasP, including local, national, international social, economic, and/or political conditions). No incentives were given for participation in this study.

### Data analysis

Interview recordings were transcribed verbatim, de-identified, and uploaded to Nvivo 10 with password protection for analysis. Transcripts were analyzed by drawing on a grounded theory approach [25]. We read and reread all the transcripts by using constant comparative techniques. As our analysis progressed, we used an open-coding approach in which coding was first organized into two broad thematic categories regarding the implementation and scale-up of TasP: “barriers” and “facilitators.” As additional interviews were completed, the open codes were grouped into conceptual categories (i.e., “trees”) related to the five CFIR constructs, with a particular emphasis on identifying the challenges and opportunities regarding the implementation and scale-up of TasP in BC and how those opportunities and challenges changed or remained stable over time. Throughout this process, we identified various recurring, converging, and contradictory themes within and across the entire data set as they related to the five CFIR constructs. Data collection and analysis occurred in an iterative fashion, whereby new interviews gathered throughout the study duration were used to further answer our analytic questions related to each CFIR construct. In doing so, we conducted both an inductive analytic approach to develop our initial coding schema and general themes, as well as deductive approaches in which our findings were used to compare and contrast CFIR constructs [25].

### Reporting standards

Reporting of this study followed the Standards for Reporting Qualitative Research (SRQR) (Additional file 1) [26].

## Results

We interviewed a total of ten participants, including eight policymakers (four at the provincial level and four at a regional health authority level, one also actively involved in providing clinical care for PLHIV) and two community-based organization representatives. Overall, participants had varying degrees of experiences with the HIV response, with around half being involved in some capacity, including as care providers and scientists/researchers, since the late 1980s and/or early 1990s in BC. Several participants indicated that they had held multiple positions across different organizations and sectors, and that the insights shared come not only from their role at the time of the interview but also from the other roles they previously held.

Below, we present our findings organized by CFIR domains, recognizing that these are not mutually exclusive categories [27].

### Intervention characteristics: “Are you reaching people, are you testing them, are you diagnosing them, are you getting them on treatment?”

Participants described how key attributes of the TasP intervention influenced the success of scaling up TasP in BC. Some described how they felt the quality and validity of evidence that provided empirical support of a positive association for TasP and health in the mid to late 2000s may have influenced stakeholder “buy-in” at multiple levels. For example, several participants described how learning about the various emerging intervention characteristics of TasP, including the individual and population-level features as well as cost-benefits, made the approach compelling to some political audiences:

*Generally speaking there’s three things that in this modern day, that the health system looks for. Is it going to be a benefit to individuals? Are you going to have an individual health benefit? Is it going to have a population level health benefit? And is it going to avert costs? […] And you know, this [the case for TasP] checked off all the boxes. (Participant 8, policymaker)*

Several emphasized that the perceptions of key stakeholders regarding the evidence supporting TasP as a treatment framework required an “ontological shift”—of sorts—from thinking about treatment as an interest for individual patients to also featuring the interests of the population. Several participants explained how this shift in thinking influenced how various implementation decisions were made early on, including the development of monitoring frameworks and systems that have the capacity to measure population-level outcomes related to the various programs. For example, one participant described how the shift to treating HIV from a public health perspective that also featured the importance of prevention shaped how they evaluated the various scale-up efforts:

*I think that the fact that the executive sponsor was the VP of Public Health brought to this project was that we could continue to advocate for and eventually insist on a population-level approach. […] And I think that was really important in hindsight because it allowed us to put a monitoring framework in place which meant that we started looking at every single thing we did in our monitoring framework. […] Everything we funded including internal and external programs were continuously monitored according to the goals of: Are you reaching people, are you testing them, are you diagnosing them, are you getting them on treatment? (Participant 5, policymaker)*

Despite the growing body of evidence supporting TasP, several participants described how there were significant concerns among affected communities (e.g., PLHIV) and the health care providers tasked with implementing TasP (e.g., both generalists and HIV specialists). For example, several policymakers described that a key challenge for rolling out TasP was in presenting the approach as having both individual- and population-level benefits. For instance, the perceived difficulty of the intervention, reflected by concerns about the radicalness and disruptiveness of the approach, were frequently surfaced as primary concerns early on. As one participant described:

*There was certainly a fear that people had that somehow we weren’t going to be treating people for treatment for their sake, but we were going to be treating people for the sake of preventing transmission. And, so, I think it was always a struggle to communicate the fact that that wasn’t the case, that we weren’t – this wasn’t about mandating people to be on treatment to prevent transmission. This was about better reaching and engaging, you know, confirming that there is a secondary prevention benefit to treatment. (Participant 8, policymaker)*

Another concern was around how TasP was not sufficiently designed to address the social determinants of health. In light of these concerns being raised, several participants described how they refined program goals and evaluation metrics within their portfolio that could also assess social and HIV-related vulnerabilities:

*There was a lot of chitter chatter at the beginning about, ‘but you’re not addressing the social determinants of health and you’re not doing this and not doing that.’ […] So one of the things that this project allowed us to do was to actually define goals and achieve those goals with a population health level lens level with a social determinants of health lens on it absolutely addressing the vulnerabilities, the ethical issues and all that but keeping your eye on the prize. (Participant 5, policymaker)*

### Inner setting: “concerns about how resources would be distributed” and “opportunity to create this integrated system”

Several participants described that the relatively large investment to the implementation and ongoing operations of TasP influenced how they perceived the importance of TasP going forward, including with regard to training, education, and reallocating intervention resources (e.g., universal ART)—all of which indicated that the government was serious about “doing things differently” with regard to HIV. For example, one participant described:

*It [the financial investments by the Province] didn’t pay for everything but it was enough money to really capture people’s attention and send a message to senior folks in health authorities responsible: ‘This is really important to government, we’re putting some money behind it and it’s a bit unusual, work with us.’ (Participant 6, policymaker)*

Nevertheless, several participants described how features of the inner setting, such as the existing networks of stakeholders and approach to communicating within and across various facets of the health system, needed to be adapted to sufficiently respond to community concerns that were being raised, including with respect to the social determinants of health. For example, one participant from a community-based organization identified that their organization had concerns about how resources would be distributed between and across non-governmental organizations, given that they viewed TasP as largely a biomedical approach to HIV intervention being adopted:

*There was funding put towards, you know, approaching HIV in a very systematic way about a community viral load […] The problems came, you know, from our perspective was more about how it was a completely biomedical approach as opposed to the social determinants being considered. Or, you know, resources put towards dealing with the social determinants as well as the biomedical. (Participant 4, community-based organization representative)*

The concerns of the community emerged as an important feature of the inner setting implementation climate, and several participants described how they had spent significant resources in trying to address these concerns. For example, one participant described how they had engaged with various strategies to address community concerns:

*STOP did not happen from one day to another. It was announced one day, but it took from 2006 to 2010 to work around engaging communities, and we had several town hall meetings with community groups we reached out. I met with them one-on-one. I met with the boards. I went to their meetings. We had conversations. (Participant 7, policymaker)*

Participants also described how structural characteristics, particularly the social architecture of service and health care providers, also needed to be adapted to produce a more fulsomely coordinated and holistic service delivery system. Despite increased funding, implementation burden on service and health care providers was described by participants to have continued during scale-up of TasP. Policymakers described their growing awareness of the need to integrate services and redesign the system. They described how silos were removed to facilitate collaboration and increase efficiency in service delivery. One participant noted that careful stewardship with multiple stakeholders was critical for the development and coordination of better integrated systems:

*After we put six million dollars of new services into the system [and] are still feeling like completely burdened, then we have to look at how we are organizing our services. […] We took that as an opportunity to really kind of create this integrated system, and so it was sort of just very thoughtful. (Participant 9, policymaker)*

### Outer setting: “compelling arguments” and “soft levers”

Interview participants described their perspectives on the external policies and incentives that influenced initial and sustained investments in the implementation and scale-up of TasP in BC. Emerging epidemiological evidence (2000–2005 approximately) regarding the economic feasibility of TasP grew alongside a broad political desire to design government policy that reduced costs to the health system, including projected long-term costs associated with the provincial HIV epidemic [28, 29]. Therefore, several participants described that TasP-related policies were viewed by decision-makers as being sustainable and cost-saving. For example, one participant described:

*The situation the government was faced with was a growing cost to provide HIV medications and to care for PLHIV was going up every year. And so essentially what, I think, [the case for TasP] was able to provide a compelling argument is to say, “Look, if you make this added investment now, we can finally get ahead of the curves.” (Participant 8, policymaker)*

As such, outer setting policies that were external to TasP but influenced how TasP was perceived (e.g., politically, socially) were described as being important factors that influenced the overall acceptability and feasibility of TasP. For example, participants described how the political and health policy “landscape” during the early and mid 2000s in BC had become amenable to advancing interventions that addressed the needs of historically stigmatized populations, including people who use drugs. Participants described how the BC government at the time, despite its socially and fiscally conservative underpinnings, acknowledged issues around substance use harms in the province and worked to address them through policy responses that included harm reduction:

*I mean, this is the same government that championed supervised injection, allowed for heroin trials. Like they’re clearly, yes, a right-of-centre government, sure, but already a pretty long track record of being interested in thinking through policy responses related to problematic substance use and communicable diseases that were disproportionately affecting people who use drugs. […] I’m not surprised the government made this decision, because there it was aligned with a lot of the… with other similar bold policy moves [in areas of] problematic substance use (Participant 8, policymaker)*

One participant described how, given the political climate at the time, it was possible to use “soft levers” (i.e., measures that are voluntary and non-binding) such as policy guidelines and recommendations on substance use and mental health to bring attention to and advance harm reduction efforts, and that this created important opportunities to gain political buy-in for TasP and implement new “systems-level” approaches to HIV intervention at that particular time in BC:

*It was very clear that harm reduction was a front and center element of all of our response to both drug use and addictions and to HIV issues. […] You know it never got to the sort of heavy sort of hammer of legislative or enforced options but we certainly were using all the kind of soft levers that we had available in terms of meetings and policy guidance documents and you know really working with the health authorities to try to deal with some of those issues. […] The political buy-in for TasP, I think it again stems originally to the political leadership […] Harm reduction was a core element of provincial policy. (Participant 5, policymaker)*

### Characteristics of individuals: “intangible but invaluable”

Several participants described how the personal traits, intellectual curiosity, motivation, values, and competencies of key decision-makers and opinion leaders were crucial to shaping the implementation and scale-up of TasP in BC. For example, one participant described how their enthusiasm for learning and open mindedness allowed them to embrace a fresh approach to addressing the HIV epidemic. These personal traits and attitudes were attributed as an opportunity for TasP acceptability and implementation:

*I kind of showed up bright eyed and bushy tailed saying ‘Gee, I wonder what I could learn’ kind of thing. And I think that was an opportunity in hindsight because I had no preconceived notions. And very quickly, I sort of realized that the opportunity, this opportunity could very quickly dissipate into no effect whatsoever if we just divide the money into existing programs which could have been very easily done and been entirely justified cause a lot of those programs needed funds. (Participant 5, policymaker)*

Another participant described the importance of the personal attributes of some implementation influencers, including a team comprised individuals with a commitment to serving marginalized communities and extensive expertise and interest in addressing the HIV epidemic:

*I had some fabulous people working on my team […] It’s a big subset of the [leadership group], both of the policy and on the program and operational side, to having worked in our misspent youth at places like [local HIV/AIDS service organization]. […] So it was not only, um, just sort of like your standard group of folks working in the Health Authority or the Ministry [of Health], it was a particularly energized group of folks who had demonstrated across you know a decade and a half in different roles, that HIV in particular was something that they felt pretty strongly about […] So there’s something in that that is intangible but invaluable. (Participant 6, policymaker)*

While senior leadership involved in the implementation of TasP were described to have been confident in their capabilities to plan and execute the implementation of TasP, at times during our analysis, the extent to which others explicitly questioned the values and motivations of senior leadership emerged throughout our interviews as an important sub-theme. For example, participants described feeling that the motivations of the various senior-level actors were not “altruistic” or underpinned by values within public health, but rather served as a means to attain a “legacy” for those who could later trademark and make intellectual claims to the approach. For example, one participant described:

*Here’s an opportunity for us to contribute that kind of thing that politicians love to be a little bit part of, a kind of a legacy with a capital “L” and while it won’t be a front and center legacy cause other people are forging along, it’ll be British Columbia’s sort of little pride to be contributing to something. So, so I think that was really important and partly that was about work that [the BC Centre for Excellence in HIV/AIDS] did to kind of really, um, explain that, explain the opportunity to contribute internationally. (Participant 6, policymaker)*

For some “middle-level influencers” (e.g., program managers or directors), reconciling how to be involved with TasP when they were explicitly questioning the values and motivations of those that they perceived as chasing legacy over “doing the right thing” emerged as an important sub-theme. For these participants, being involved became especially important, as they wanted to ensure that the TasP enterprise could be designed in ways that were social justice-oriented—something they viewed as being unable to transpire under the “top-down” approaches that some individuals wanted and tried to advance. As such, several participants highlighted how, despite their serious reservations and doubts about the senior leaderships’ trustworthiness and motivations, their own strong commitments to “doing the right thing” galvanized their dedication to being involved in order to make sure the successful implementation and scale-up of TasP would feature commitments to social justice and vulnerable populations. As one participant described:

*And it’s not just because we’re paid to do this, it’s because we’re passionate because we, many of us and particularly myself, have worked with a variety of populations from very disadvantaged in the Downtown Eastside [local drug scene area with high HIV rates] to our current clinic in highly stigmatized with transgender populations, with gay populations, […] We knew that there was enough evidence, not perfect, not conclusive, but enough evidence for us to rally around creating that opportunity. […] I think we created the opportunity to ourselves and most importantly to the population that we served. (Participant 7, policymaker)*

### Process: “we listen to the concerns, we address them”

Participants described how the processes (planning, engaging, executing, and reflecting and evaluating) associated with the scale-up of TasP were designed in such a way that they could be highly responsive and adaptable to “real time” data. For example, one participant described how the development of population-level monitoring and evaluation systems allowed for feedback loops that enabled the ability to make informed decisions to scale up routine testing and adapt the system:

*We have population-level monitoring […] And what that allowed us to do is to not take undue credit just because we opened a clinic that tested a few thousand people, because if those people were gonna be tested somewhere else then we haven’t achieved anything. And so we did a year of shoring up existing programs. We did a population-level intervention and nothing changed and that’s why we moved to routine testing. We moved to routine testing because of that monitoring and evaluation framework was telling us that what we were doing wasn’t making any difference. (Participant 5, policymaker)*

Scaling up TasP across BC’s highly decentralized service delivery system (e.g. multiple health authorities) was, at times, described as a key opportunity for doing things differently. For example:

*There’s very little sort of consistency in the health system in BC. […] I mean it’s partly a problem, it’s partly also a positive in it allows for innovation and sort of experimentation and you know those can be really good things and so, you know, if you use part of the top down mandate everybody shall do this and that’s what they do then you know you don’t necessarily get new ideas gurgling up because somebody’s willing to take a risk here where others aren’t. (Participant 5, policymaker)*

As such, there were several instances in which participants described how they were able to develop processes during scale-up of TasP that facilitated the bridging of HIV services with other facets of the health care service delivery system. For example, peer navigation services were added to facilitate clients’ access to needed services, including health care, harm reduction, and housing services:

*We have created this very highly complex system where people – to get care, they really need to navigate different layers and know where to go and quite often you end up going to the wrong place […] So we thought that peer navigation plus outreach where we help to overcome some of these barriers. And so specifically for the Downtown Eastside […] pairing up methadone, this daily dispensing with antiretroviral therapy and mental health or psychiatric medications with creating opportunities for HIV dedicated housing for people with high complex conditions particularly dual diagnosis and so on. (Participant 7, policymaker)*

Despite the success participants associated with the scale-up of TasP, the distrust some expressed towards senior leadership was described as having a negative knock-on effect onto the overall effectiveness of many of the processes associated with planning, executing, and evaluating the scale-up of TasP. For example, a few participants suggested that, at times, senior leadership had a level of executive procedural power over key policy decisions and described that this was particularly challenging given they did not always trust the motivations of leadership. For example, one participant described how they questioned the motivations of senior leadership, particularly when program concerns were not considered to be sufficiently addressed, thereby resulting in “top-down” decision-making processes:

*What motivated it [i.e., launch of STOP HIV/AIDS] was [senior leadership] saw that the number of HIV folks were decreasing, which meant that the budget would decrease, so [they] wanted to make sure that everyone in the province was tested for HIV so the empire continued. So [they] created a sense of urgency around HIV and numbers which still haven’t actually been born out, where we were testing to beat cases out of [local hospital] that weren’t there […] We were told to stop questioning the value of routine testing versus enhanced testing for priority groups […] If we couldn’t get behind it, we were told to get out of the way. It was not a place where you were actually allowed to discuss and debate policy. (Participant 2, policymaker)*

## Discussion

To our knowledge, this is the first study to apply CFIR to explore key opportunities and challenges that influenced the implementation and scale-up of TasP. Specifically, we identified the key implementation opportunities associated with each CFIR construct, including the following: (i) factors that enhanced stakeholder buy-in based on features of the *intervention characteristics*, including with regard to assessments about the quality and strength of evidence supporting TasP; (ii) an *inner setting* implementation climate that was, in part, shaped by the large and highly symbolic government investments into TasP; (iii) features of the *outer setting* such as external policies (e.g., harm reduction) that cultivated opportunities to implement new systems-level approaches to HIV intervention; (iv) the personal attributes of some “middle-level” influencers, including a team that was comprised of some highly motivated and social justice-oriented individuals (e.g., folks who were deeply committed to serving marginalized populations); and (v) the capacity to develop various *implementation processes* that could maintain “nimble and evidence-informed” adaptations across a highly decentralized service delivery system, while also creating opportunities to adapt features of TasP programming based on “real time” program data.

Based on the CFIR domains, we also identified areas where specific implementation challenges played out, including the following: (i) stakeholder concerns about the appropriateness of various features of the *intervention characteristics* (e.g., concerns that routine testing was not an effective use of resources); (ii) concerns about the *inner and outer settings*, including that the implementation climate was not sufficiently poised to create systems that sufficiently addressed structural concerns, including with regard to the social determinants of health and provider burden; (iii) concerns about the *individual characteristics*, including personal attributes of senior leadership that led some to question the values and motivations of their decisions; and (iv) a corresponding set of concerns that some of the *implementation processes* (e.g., engaging, decision-making) were “top-down.”

Our findings provide key tangible factors that are critical to the successful implementation and scale-up of a systems-level intervention, including those associated with characteristics of individuals and implementation process domains. The involvement of implementation influencers dedicated to serving marginalized populations appears to be instrumental to the success of various aspects of TasP implementation, including ensuring that TasP featured commitments to social justice. Policymakers and care providers committed to health equity for key affected populations, including those who had previously dedicated careers to serving gay, queer, and trans populations affected by HIV, were critical in bringing a wealth of substantive and institutional knowledge about HIV, community needs, and competing priorities, in addition to a passion and energy involved in systems-level change and practice transformation. These findings align with previous research indicating that public health programs can be enhanced during scale-up when key stakeholders are involved as implementation influencers [30]. And, while our interviews revealed that some internal implementation leaders (i.e., senior leadership) missed opportunities to engage the community, those who found themselves embedded in the “new” system (e.g., middle-level implementation influencers) described being able to make changes that more appropriately responded to community needs via other strategies, including those that they described as “soft levers” (e.g., internal policies).

Findings from our work underscore the importance of how key social and political features of the implementation context have a significant influence on scale-up. For example, the capacity to influence soft levers to advance harm reduction made it more feasible to implement new systems-level approaches to HIV. The implementation of soft policy measures also enhanced the overall acceptability of “doing things differently” within and across health care delivery systems to address the HIV epidemic—something reinforced through provincial support for harm reduction measures. And, while BC government’s initial investment of $48 million into STOP HIV/AIDS demonstrated a commitment and received eventual and widespread support across the system, the involvement of community and community activist efforts continued to inform many of the soft levers identified within the current study.

To our knowledge, this is the first study to identify how these kinds of soft levers may have profound influence in the implementation of systems-level changes in HIV treatment and prevention. While literature on systems levers for primary mental health care has identified how soft levers such as engagement (i.e., activities to foster and maintain relationships and dialogue among stakeholders) can influence health system change, soft levers identified in our study and the mechanisms through which they influence systems-level changes have yet to be documented in health literature [31], thereby indicating that future research in this area is warranted.

Finally, our findings elucidated the importance of developing capacity to adapt features of TasP programming based on emerging data and evidence during implementation and scale-up. This is in line with previous research, which has suggested that monitoring and evaluating TasP implementation efforts is critical to enhancing the efficiency and effectiveness of the intervention and optimizing health outcomes [32]. In BC, incorporating real-time, population-level monitoring and evaluation systems were critical to identifying key challenges and opportunities, and enabling TasP to be highly responsive throughout much of the implementation process.

### Strengths and limitations

This study has several strengths and limitations. While our sample size is small, findings from this study are not intended to be generalizable. The findings nevertheless offer critical insights that may be used to inform HIV treatment and prevention efforts and implementation science in other settings. Our study is among the first to document the key factors that influenced the implementation, adaptation, and scale-up of TasP, an outcome that provides a unique and valuable contribution to both the HIV and implementation science literature. However, these findings need to be interpreted with caution. For example, there are several limitations to this study design. Four people invited to participate in the study were unable to be interviewed due to time constraints and scheduling conflicts. Thus, valuable perspectives may have been missed in our analysis. Furthermore, despite assurance of privacy and confidentiality, social desirability bias may have affected participants’ willingness to share certain experiences and attitudes, particularly as they relate to communities, organizations, and individuals involved in TasP implementation and scale-up.

## Conclusion

This study identified key implementation opportunities and challenges that influenced the implementation and scale-up of TasP in BC. Constructs across all five domains of CFIR (intervention characteristics, outer setting, inner setting, characteristics of individuals, and process) were identified to influence the success of TasP. Our findings have implications for how BC can successfully implement and scale up other systems-level interventions that have demonstrated efficacy and offer important insights for other jurisdictions that are currently or plan to scale up TasP.

## Supplementary information

**Additional file 1.** Standards for Reporting Qualitative Research (SRQR) Checklist.

## Data Availability

All relevant data are presented within the paper and are fully sufficient to replicate the study findings.

## Abbreviations

BC: British Columbia
TasP: Treatment as Prevention
CFIR: Consolidated Framework for Implementation Research
ART: Antiretroviral therapy
PARTNER: Partners of People on ART—A New Evaluation of the Risks
START: Strategic Timing of Antiretroviral Therapy
U=U: Undetectable equals untransmittable
PLHIV: People living with HIV
STOP HIV/AIDS: Seek and Treat for Optimal Prevention of HIV/AIDS
